# Perinatal health care services for imprisoned pregnant women and associated outcomes: a systematic review

**DOI:** 10.1186/s12884-016-1080-z

**Published:** 2016-09-29

**Authors:** Eleanor Bard, Marian Knight, Emma Plugge

**Affiliations:** 1 Oxford University Hospitals NHS Trust/Nuffield Department of Population Health, University of Oxford, Old Road Campus, Roosevelt Drive, Oxford, OX3 7LF UK; 2 National Perinatal Epidemiology Unit (NPEU), University of Oxford, Old Road Campus, Oxford, OX3 7LF UK; 3 Centre for Tropical Medicine and Global Health, Nuffield Department of Clinical Medicine, University of Oxford, Old Road Campus, Oxford, OX3 7FZ UK

**Keywords:** Prison, Prisoner, Pregnant women, Pregnancy outcomes, Antenatal care, Perinatal care, Postnatal care

## Abstract

**Background:**

Women are an increasing minority of prisoners worldwide, and most are of childbearing age. Prisons offer unique opportunities for improving the pregnancy outcomes of these high-risk women, and no systematic review to date has looked at their care. This systematic review identified studies describing models of perinatal health care for imprisoned women which report maternal and child health and care outcomes.

**Methods:**

We systematically searched for literature published between 1980 and April 2014. Studies were eligible if they included a group of imprisoned pregnant women, a description of perinatal health care and any maternal or infant health or care outcomes. Two authors independently extracted data. We described relevant outcomes in prisons (including jails) under models of care we termed *PRISON*, *PRISON+* and *PRISON++,* depending on the care provided. Where outcomes were available on a comparison group of women, we calculated odds ratios with 95 % confidence intervals.

**Results:**

Eighteen studies were reported, comprising 2001 imprisoned pregnant women. Fifteen were in the US, two in the UK and one in Germany. Nine contained a comparison group of women comprising 849 pregnant women. Study quality was variable and outcome reporting was inconsistent. There was some evidence that women in prisons receiving enhanced prison care, *PRISON+*, were less likely to have inadequate prenatal care (15.4 % vs 30.7 %, *p* < 0 · 001), preterm delivery (6.4 % vs 19.0 %, *p* = 0 · 001) or caesarean delivery (12.9 % vs 26.5 %, *p* = 0 · 005) compared to women in prisons receiving usual care (*PRISON)*. Women participating in two *PRISON++* interventions, that is, interventions which included not only enhanced care in prisons but also coordination of community care on release, demonstrated reductions in long term recidivism rates (summary OR 0 · 37, 95 % CI 0 · 19–0 · 70) compared to pregnant women in the same prisons who did not participate in the intervention.

**Conclusions:**

Enhanced perinatal care can improve both short and long-term outcomes but there is a lack of data. Properly designed programmes with rigorous evaluation are needed to address the needs of this vulnerable population. The cost to mothers, children and to society of failing to address these important public health issues are likely to be substantial.

**Trial registration:**

PROSPERO registration: CRD42012002384.

## Background

Women are a small but increasing minority of the 10 · 2 million people imprisoned worldwide [1]. There are around 100 000 women in prison in Europe on any 1 day, representing 5 % of the total prison population [2]. In the United States (US) there are nearly 215 000 women in prisons and jails, representing 9 % of the incarcerated population and an absolute increase of 30 % since 2000 [3]. Despite growing numbers, women’s minority status means that their specific health care needs and those of their children may be overlooked or remain unmet. A review from the United States found that 38 states had inadequate or no prenatal care in their prisons [4], and a 2008 report from US Department of Justice notes that 46 % of pregnant imprisoned women reported they received no pregnancy care [5]. The World Health Organisation’s (WHO) 2003 Moscow declaration recognises prison health as an important public health issue [6], and a 2009 WHO declaration acknowledges that current arrangements for dealing with women offenders often fail to meet their basic and health needs and specifically mentions inadequacies in provision for imprisoned pregnant women [2]. The 2010 United Nations Bangkok rules [7] and the 2015 Standard Minimum rules for the Treatment of Prisoners [8] provide guidance on perinatal care in correctional settings and state that pregnant women should be provided with a healthy environment and the same standards of health care that are available in the community. The provision of adequate perinatal care in prison is also the law in the US under the Eighth Amendment which prohibits “cruel and unusual punishment.”

As the number of women in prison grows, so does the number of imprisoned pregnant women and mothers; most imprisoned women are of childbearing age and an estimated 6 % are pregnant, although there is no recent or accurate statistic establishing this proportion [9]. However, although imprisoned pregnant women are at high risk of poor perinatal outcomes due to factors such as ethnicity, low levels of education, access to antenatal care, smoking, drinking alcohol and illegal drug habits [10], a review of perinatal health care in prisons found that there was a lack of available data on perinatal health care worldwide [11]. In a synthesis of the limited evidence available in 2005 we found that imprisoned pregnant women had poorer outcomes of pregnancy than the general population but better outcomes than similarly disadvantaged groups of women not imprisoned [12]. The former finding highlights this group as a vulnerable population worthy of further investigation. The latter demonstrates that prisons offer unique opportunities for improving the health care and pregnancy outcomes of a group of high-risk women when they need to be imprisoned, contributing to the health of both mother and child in the short and longer term; particularly given the growing evidence that events during early development, including the foetal period, have a profound impact on one's risk for development of future adult disease [13].

The health care provided to imprisoned pregnant women is of considerable public health importance, and no systematic review to date has looked at this care. This study aimed to identify effective models of care for these women. The specific review objectives were: to describe models of perinatal health care for imprisoned women which exist in the research literature and subsequent maternal and child health and care outcomes; and to examine, where possible, the effectiveness of models of perinatal health care for imprisoned women on subsequent maternal and child health and care outcomes.

## Methods

### Search strategy and selection criteria

We developed a protocol for the systematic review using PRISMA guidelines [14], which was prospectively registered in the PROSPERO database (International Prospective Register of Systematic Reviews), registry number CRD42012002384.

We searched Medline, Embase, PsycINFO, Global Health, CINAHL, The Cochrane Library database, Scopus, Web of Science, Applied Social Sciences Abstracts (ASSIA), Campbell Collaboration (C2-Spectr and C2-RIPE), CareDATA (Social Care Online), Health Management Information Consortium (HMIC), Intute (previously SOSIG) and the National Criminal Justice Reference Service Abstracts to identify relevant articles, searching from 1980 to April 2014. Search terms (Table 1) were identified from database thesauri, and included prisons and jails. The terms were combined within columns using the “or” operator, and between columns using the “and” operator.

**Table 1:** Key words and Medical Subject Headings (MeSH) used in literature search

Population	Intervention
MeSH terms:	MeSH terms:
Pregnancy	Prison
Pregnancy outcome	Prisoner
Pregnant women	
Pregnancy complications	Free text words:
Prenatal care	Prison*
Perinatal care	Gaol*
Postnatal care	Jail*
Neonatal care	Incarcerat*
Birth/Delivery/Childbirth/Parturition	Imprison*
Postpartum period/puerperium	Penal
	Penitentiar*
Free text words:	Correctional facilit*
Pregnan*	Correctional institution*
Antenatal	Justice facilit*
Prenatal	Justice institution*
Perinatal	Inmate*
Birth*	Detain*
Parturition*	Detention*
Childbirth*	Offend*
Postnatal	
Postpartum	
Puerperium	
Neonatal	

*The search will retrieve variations on the word stem preceding

Electronic database searches were supplemented with hand searches of the references of selected papers and relevant policy documents. We undertook extensive but targeted grey literature searching including contacting relevant prison health-related networks.

Throughout this paper, the term “imprisoned” is used for simplicity but includes women incarcerated in jails and prisons.

### Data extraction

After removal of duplicates, we screened all abstracts and obtained full manuscripts of all possible eligible citations, irrespective of language. EB and EP independently assessed these manuscripts for inclusion using pre-specified criteria (Table 2) and then independently extracted data from included studies using a proforma. MK mediated any disagreements related to eligibility, risk of bias, or data. Authors were contacted if further information was required. Data, extracted from papers using a pre-prepared proforma, included language, publication date, study design, setting, study duration and dates, details of participants, care received by participants, control selection, source of outcome measurement, outcomes, results of study, and funding source. The risk of bias was assessed for each paper as part of the data extraction process with a domain-based assessment adapted from Cochrane Database guidelines [15]. Risk of bias for various components of the studies was classified as high, low or unclear.

**Table 2:** Eligibility criteria

Inclusion criteria		Exclusion criteria
Population	Inmates	Concentration camps/non-penal institutions
	Women	Men
	Any age	Asylum seekers in detention
	General prison population	Previous inmates
	Imprisoned at some point during pregnancy, up to and including delivery	Never pregnant during imprisonment
		Psychiatric units
Intervention	Model of perinatal health care described^b^	No description of perinatal health care
Control group	No comparison group needed	
Outcomes	Pre-specified perinatal outcomes quantified^a^	No pre-specified perinatal outcomes quantified
	Early childhood outcomes quantified (up to age 5)	No early childhood outcomes quantified (up to age 5)
	Any other measures of maternal morbidity quantified	No measures of maternal morbidity quantified
	Outcomes relating to health care utilisation quantified	No such outcomes measured
Studies	All languages	
	Data collected after 1980	Data collected before 1980
	Any study design	

^a^Pre-specified perinatal outcomes were: miscarriage, fetal anomaly, preterm delivery, small for gestational age, low birthweight, mean birthweight, stillbirth, perinatal death, neonatal death, infant death, admission to neonatal intensive care, breastfeeding, caesarean section rates, and instrumental delivery rates

^b^This included any description of perinatal health care, however minimal

### Data synthesis

We described all relevant outcomes across studies to present a picture of perinatal outcomes in prison under different models of perinatal health care. Using the descriptive data provided in the text, we classified the care in the intervention group of imprisoned women into three levels of care according to the services they received. *PRISON* described models of perinatal health care that represented usual care for that prison with no attempt having been made to improve perinatal health care or implement any intervention. *PRISON+* described models of perinatal health care where some specific effort had been made to improve conditions or care for pregnant prisoners. Women receiving *PRISON++* care are provided with alternative accommodation during pregnancy and co-residence with their children after birth, with strong links between these programmes and community services, recognising that the support for women must continue after release from prison. Where possible we examined differences in outcomes between *PRISON, PRISON+* and *PRISON++ groups.*

Where outcome data were available on women or their babies in either of the three intervention groups described above and a comparison group of women receiving an alternative model of care, we calculated odds ratios or weighted mean differences with 95 % confidence intervals using fixed effects models (Mantel-Haenszel) or random effects models if there was evidence of significant heterogeneity between groups (evidenced by the I^2^ statistic). Summary measures for odds ratios comparing outcomes in the intervention groups with comparison groups were calculated and presented where appropriate. We categorised comparison groups into disadvantaged controls: those experiencing similar social disadvantage to imprisoned women, through drug use, previous criminal conviction or imprisonment; and population controls: those selected from a general population in whom no such factors were identified. The data were analysed and presented with STATA (version 12). Forest plots were produced for the available perinatal outcomes, stratified by type of comparison group.

## Results

**Fig. 1:**
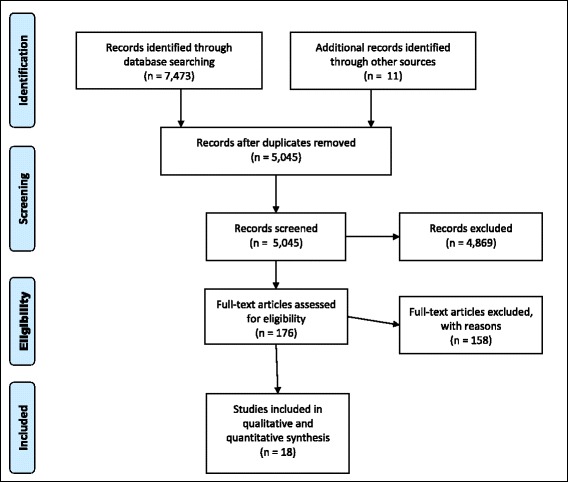
Study Selection

From a total of 7484 studies found through systematic and grey literature searching, we assessed 176 full-text articles for eligibility (Fig. 1). Eighteen of these were eligible for inclusion (Table 3) [16–33], comprising a total of 2001 imprisoned pregnant women. Seventeen studies were written in English and one in German; 15 were conducted in the US [16–24, 26, 28–32], two in the UK [25, 27], and one in Germany [33]. Of these, nine contained a comparison group which enabled us to compare outcomes in the intervention groups and comparison groups [16, 17, 19, 23, 24, 26, 31–33], which comprised 472 population controls and 377 disadvantaged controls. Summary of the risk of bias by domains is shown in Table 4.

**Table 3:** Descriptive summary of included studies

Reference	Setting	Study design	Participants (imprisoned pregnant women and control women)	Outcomes	Description of care in intervention group
*PRISON* category					
Bell 2004 [17]	King County Jail, Seattle, Washington, US	Cohort. Disadvantaged comparison group.	Intervention group: 468 women imprisoned while pregnant in County jail delivering live, singleton births in the state 1994–1998. Disadvantaged comparison group:144 women delivering live, singleton births in the state 1994–1998 who spent time in jail at a time other than during pregnancy. They received community care coordination throughout pregnancy and one year postpartum.	Perinatal health service use: inadequate prenatal care, Maternity Support Service use, Maternity Case Management use, any family planning services post-birth, any prenatal care use, no prenatal care use	On-site health clinic staffed by local health department. Prenatal care offered on arrival to jail. Women in jail are not eligible for Maternity Support Services or Maternity Case Management. Those with history of substance abuse referred for post-release case management.
Kyei-Aboagye 2000 [24]	Two Correctional Facilities, Massachussetts, US	Cohort. Population and disadvantaged comparison groups.	Intervention group: 31 women imprisoned at 2 facilities during 2nd trimester of pregnancy delivering at one hospital between 1993 and 1996. Population comparison group: 71 unmatched randomly chosen non-imprisoned women delivering at the same hospital. Disadvantaged comparison group: 47 women enrolled in a methadone maintenance programme.	Low birthweight, mean birthweight, APGAR scores	Boston Medical Center signed agreement in 1993 to provide obstetric care at 2 facilities. All basic prenatal care provided in jail by obstetricians and nurse practitioner or registered nurse, with transfer to Boston Medical Center for delivery and if needed at other times.
Leifer 2003 [25]	Holloway Prison, London, UK	Case series. No comparison group.	120 pregnant prisoners who delivered at the Whittington Hospital up to December 2002.	Mean birthweight, stillbirth, neonatal unit admission	Since 1998 maternity services provided by Whittington Hospital. Escorted to hospital for birth; officers leave unless security risk. Detoxification 12–28 weeks if necessary. Progress recorded in maternity notes, used by paediatric staff at Whittington and by social services to prove detoxification. Can apply to mother and baby unit.
Mertens 2001 [26]	County Jail, Illinois, US	Cohort. Population comparison group.	Intervention group: 72 pregnant women imprisoned in a county jail in one calendar year. Population comparison group: 52 pregnant women identified from state records and matched by age, race, gravidity and zip code of residence.	Miscarriage, low birthweight, stillbirth (cases), inadequate prenatal care	Pregnancy test and examination on admission. Health services provided by family practice physician and registered nurses. One full-time counsellor for whole women’s unit, no nutrition services, periodic head counts meant missed prenatal appointments.
Shelton 1989 [30]	Missouri, Maryland, US	Case series. No comparison group.	26 English-speaking women in last trimester of pregnancy in two women’s correctional centres with expected deliveries May – December 1982 while still imprisoned.	Preterm labour, neonatal unit admission, caesarean, pregnancy complications, newborn complications, reproductive tract infections, hypertension, diabetes, varicosities, uterine dysfunction, breech presentation, placenta praevia, placental abruption, incompetent cervical os, cephalopelvic disproportion, first trimester bleeding, iron deficiency anaemia, postpartum depression	One facility: health centre nurse held sick call each day. Appointments made with obstetrician for the day they monitor obstetric patients at County Health Department. Private doctor attends once per week and provides for obstetric emergencies only if present when happens. Other facility: from 8th month to one month post-partum, women admitted to intake area in prison hospital. Sick call every day with nurse. Transferred to hospital if needed or referred to physician who attended regularly.
Stauber 1984 [33]	Berlin Prisons, Germany	Cohort. Population comparison group.	Intervention group: 43 women from Berlin Prisons who delivered babies at Charlottenburg University Hospital 1973–1982. Population comparison group: 172 women matched with cases by age, parity and marital status who delivered babies at the same hospital.	Preterm delivery, small for gestational age, congenital anomaly, postpartum asphyxia, for adoption, breastfeeding, placental retention, postpartum haemorrhage, congenital syphilis, heroin withdrawal, umbilical cord infection.	Illness or complications related to pregnancy treated by women’s clinic in Charlottenburg which also provides prenatal testing and ultrasound. Basic prenatal care provided at prison site by midwives and gynaecologists. Some extra nutrition provided. No reduced duties or access to pregnancy-related education.
Terk 1993 [32]	Two prisons, Texas, US	Cohort. Population comparison group.	Intervention group: 76 imprisoned pregnant women who delivered January 1987 – May 1990. Population comparison group: 117 unmatched randomly chosen non-imprisoned pregnant women delivering at the same hospital in the same time period.	Low birthweight, neonatal death, preterm delivery, APGAR scores, caesarean, prolonged rupture of membranes, birthweight < 1 kg.	Prenatal care provided by University of Texas Medical Branch medical personnel at least twice per month (obstetrician + physician assistant). Patients needing hospitalisation for prenatal complications or 1–4 weeks before estimated delivery date transferred to Department of Corrections hospital unit on University of Texas Medical Branch campus.
*PRISON +* category					
Clark 2006 [20]	Multiple jails, Florida, US	Cohort. No comparison group.	515 pregnant imprisoned women in jails in 4 counties enrolled in programme between February 2002 and December 2004. 16 HIV-positive women delivered as of December 2005.	HIV test in pregnancy, HIV outcome in child	Targeted Outreach for Pregnant Women Act (TOPWA) programme uses outreach workers to identify women in jails in 4 counties for eligibility (pregnant, lack adequate prenatal care, risk of HIV-infected or substance-exposed infant). TOPWA staff advocate for incarcerated clients to receive prenatal care, HIV related services, pregnancy and HIV testing, education on prenatal care and antiretroviral therapy use. Linked to health and social services on release. Women tracked by TOPWA until birth with phone calls and visits.
Cordero 1992 [21]	Ohio Reformatory for women, State Prison, Ohio, US	Cohort. No comparison group.	233 pregnant women imprisoned in the state medium-security prison 1986–1990.	Mean birthweight, stillbirth, preterm delivery, small for gestational age, neonatal unit admission, APGAR scores, caesarean, inadequate/adequate prenatal care, number of prenatal visits	Prenatal care at prison infirmaries and Ohio State University Hospital antepartum clinic; women transferred in 3rd trimester to pre-release centre (low security near hospital); 2800 calorie diet, vitamin and iron supplements, additional 400 calorie snack until 1988; light duties only; health education classes and Lamaze classes.
Inoue 2003 [22]	Cook County Jail, Chicago, US	Case series. No comparison group.	Intervention group: 50 pregnant women imprisoned in County Jail in the programme’s first year (2001) and receiving doula services.	Caesarean, epidural rates.	Support before, during and after childbirth. Prenatal education, doula visits, support to develop birth plan. Prenatal class with doulas in last trimester. Birth companion, pictures of baby, diary to write birth narrative. Visits every day of hospital postpartum stay.
Rowles 2007 [27]	Holloway Prison, London, UK	Case series. No comparison group.	9 pregnant women and 5 postnatal women in Holloway prison between January and March 2007.	Postnatal: Breastfeeding, positive contact with programme, companion present at birth, breast counsellor visited within 24 hours of birth, supported by breast counsellor. Prenatal: aware of birth companions, wanted birth companion present at birth.	Antenatal classes, birth plans, prison visits, support by birth companion during labour and birth, hospital visits, practical assistance, breastfeeding support, community visits.
Safyer 1995 [28]	Rikers Island Jail, New York City, US	Cohort. No comparison group.	114 pregnant women imprisoned in correctional facility in 1994 who delivered at Elmhurst hospital.	Mean birthweight, caesarean, termination of pregnancy requested/obtained in prison,	Collaboration with Montefiore Medical Center and Elmhurst Medical Center. Care includes blood tests, pelvic examination, ultrasound scan, vitamins and iron; regular physician/nurse practitioner/prenatal nurse visits ; prenatal counselling and education; special diet; housed separately in 3rd trimester; birth at Elmhurst Medical Center; jail nursery after birth if eligible; drugs counselling and methadone if needed.
Schroeder 2005 [29]	Urban Jail in Seattle, Washington State, US	Case series. No comparison group.	18 women imprisoned in urban jail during 2-year period who chose to have doula support.	Mean birthweight, gestational age, satisfaction, mean APGAR scores	Primary and back-up doula meets women prior to birth to review expectations, assess knowledge of birth, teach, develop birth plan. Doula meets women at hospital for birth support, photos and birth story. Doula follows infant placement after birth through contact with social worker. Doula visits 3 days postpartum to review experience, provide info and share photos and story.
*PRISON*++ category					
Carlson 2001 [18]	Nebraska Center for Women, Prison,, US	Cohort. Disadvantaged comparison group.	Intervention group: 44 women in nursery programme Nov 1994–Nov 1999 (37 answered survey). Disadvantaged comparison group: 30 women who delivered in Nebraska Center for Women (mixed-security confinement facility) Jan 1991–Nov 1994, before the nursery programme existed. Mothers and babies separated 3 days after birth.	Recidivism, misconduct reports, mother retained custody of child after leaving, tested positive for drugs while in programme, involuntary release, sent babies home. Survey outcomes: stronger bond with child, better self-esteem, parenting classes helped, better prepared to be working mother, would do programme again, other states should have similar programmes	Prenatal, delivery and postpartum care at local hospital. Prenatal, parenting, infant care, child development, Lamaze, breastfeeding, CPR, alternative to spanking classes. Half-time work after birth for 6 months, General Educational Development classes if not already qualified, develop and coordinate community resources during imprisonment and after release, mentor programme with follow-up after release.
Carlson 2009 [19]	Nebraska Center for Women, Prison, US	Cohort. Disadvantaged comparison group.	Intervention group: 65 women in nursery programme Nov 1994–Nov 2004. Disadvantaged comparison group: 30 women who delivered in Nebraska Center for Women (mixed-security confinement facility) Jan 1991–Nov 1994, before the nursery programme existed. Mothers and babies separated 3 days after birth.	Recidivism	Prenatal, delivery and postpartum care at local hospital. Prenatal, parenting, infant care, child development, Lamaze, breastfeeding, CPR, alternative to spanking classes. Half-time work after birth for 6 months, General Educational Development classes if not already qualified, develop and coordinate community resources during imprisonment and after release, mentor programme with follow-up after release.
Barkauskas 2002 [16]	Michigan State adult corrections system, US	Case series. Disadvantaged comparison group.	Intervention group: 37 pregnant women transferred to residential programme from county jail or state prison July 1996–Dec 1998. Disadvantaged comparison group: 35 women who entered Michigan state prison pregnant Aug 1997-Aug 1998, were eligible for programme but did not participate.	Low birthweight, mean birthweight, small for gestational age, gestational age at birth, APGAR scores, caesarean, breastfeeding, meconium at birth, oxygen at birth, respiratory difficulty at delivery, discharge weight, haemoglobin, haematocrit, spontaneous delivery, episiotomy, normal amniotic fluid amount, clear amniotic fluid, blood loss.	Women and Infants at Risk programme (WIAR): Women with a substance abuse history transferred to residential programme while pregnant until 4 months postpartum. They have their own room with baby equipment provided; prenatal, family planning and childbirth education; nutritional supplements; transport and accompaniment to appointments; birth at local hospital supported by family or volunteer; bonding room for 1 month after birth; onsite childcare facility; counselling, therapy, substance abuse education, narcotics anonymous group; employment classes, arrangements for housing, aftercare, medical care and day care on release with comprehensive coordination of care.
Kubiak 2010 [23]	Michigan State Prison, US	Cohort. Disadvantaged comparison group.	Intervention group: 48 women who entered prison pregnant 1996–1998 and were transferred to the residential programme while pregnant. Disadvantaged comparison group: 36 women who entered prison pregnant 1996–1998, were eligible for programme but did not participate.	Crown Prosecution Service file on child, foster care file on child, adoption file on child, maternal rights terminated, child formally away from mother, evidence of mother as caregiver, no evidence of caregiver besides mother, informal caregiver, formal caregiver, maternal arrest post-birth, convicted post-birth, recidivism.	See above (WIAR)
Siefert 2001 [31]	Michigan State Prison, US	Case series. Disadvantaged comparison group.	Intervention group: 44 pregnant women transferred from prison to residential programme outside the prison in 1991–1995. Disadvantaged comparison group: 120 pregnant prisoners in Michigan corrections system 1987–1991, before residential programme began. Mothers and babies separated after birth.	Preterm delivery, small for gestational age, low birthweight, stillbirth, neonatal death, neonatal admission, congenital anomaly or serious delivery complication, fetal alcohol syndrome, meconium aspiration syndrome, severe intra-uterine growth retardation, hepatitis, anaemia, suspected sepsis, discharged to maternal friend, place in foster care, infants born drug-free.	See above (WIAR)

**Table 4:**
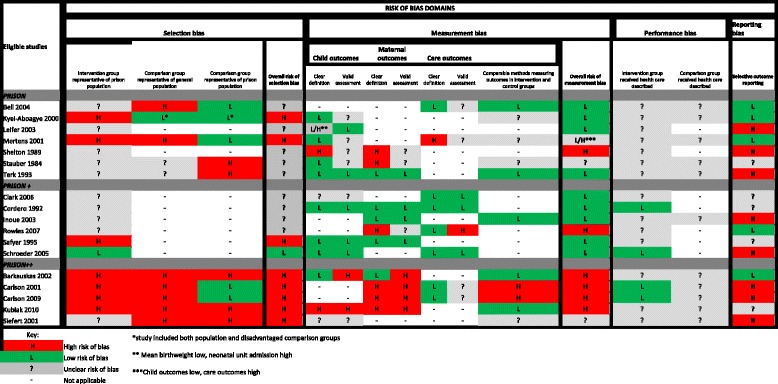
Risk of bias in eligible studies

Excluded papers either did not contain primary data on any relevant outcomes, did not contain descriptions of perinatal health care, did not include imprisoned pregnant women, or were entirely overlapping with included studies. It was notable that of 158 excluded papers, 37 (23 %) described perinatal health care programmes for imprisoned pregnant women but did not quantify any outcomes despite the fact that 35 examined specific interventions designed to improve perinatal outcomes. The care described included 15 mother and baby units within prisons [34–48], 12 prenatal/educational support programmes [49–60], four doula or birth support programmes [61–64], and six papers which described a mixture of multiple interventions [65–70]. Twenty-four were in the US [34, 39–46, 48, 49, 51, 54–56, 58, 61, 62, 64–69], eight in the UK [35–37, 47, 50, 59, 63, 70], two in France [38, 52], two in Australia [53, 60] and one in Russia [57]. Sixteen papers (10 % of those excluded) described perinatal health care programmes and quantified relevant outcomes but were not included because either the intervention occurred outside the prison setting (e.g. jail-diversion programmes for pregnant women), or because it was not clear that all women were pregnant at some point during imprisonment (e.g. nursery programmes) [71–86].

### Models of perinatal health care in included studies

Seven studies described models of perinatal health care that represented usual care in that prison: *PRISON* [17, 24–26, 30, 32, 33]*.* Antenatal care generally involved health personnel intermittently visiting the prison and transfer to nearby hospitals for birth or if complications arose. Prenatal care appointments were provided on site in four studies [17, 24, 25, 32] and it is unclear for the other three [26, 27, 33]. In one facility in Missouri women were admitted to the prison hospital from the eighth month of pregnancy until one month post-partum [30].

Six studies described models of perinatal health care where some specific effort had been made to improve conditions or care for pregnant prisoners: *PRISON+* [20–22, 27–29]. In three of these programmes, doulas/birth companions supported pregnant prisoners before, during and after birth [22, 27, 29]. Two programmes provided enhanced prenatal care for all pregnant prisoners including increased nutrition relative to other inmates, vitamins and iron supplements, reduced physical duties, prenatal counselling and education, and transfer in the third trimester to separate accommodation [21, 28]. One programme used outreach workers to identify women in jails across the state who were at risk of giving birth to an HIV-positive or substance-exposed infant and to link these women to prenatal care [20].

The five studies in the *PRISON++* category refer to two programmes in which women are provided with alternative accommodation during pregnancy, co-residence with their child after birth and are linked to community services [16, 18, 19, 23, 31]. Two studies examined outcomes associated with a live-in nursery within a women’s prison in Nebraska, US [18, 19]. Women were transferred to the nursery 1 to 2 months before birth and their babies were able to stay with them after birth. The programme provided prenatal parenting, infant care and child development education, hands-on training, and coordinated community resources available for the mother during her prison stay and upon her release. The other three studies examined a programme in Michigan, US, named Women and Infants at Risk (WIAR) [16, 23, 31]. Imprisoned pregnant women with a history of substance abuse were transferred to a residential programme outside prison where they were supported through pregnancy, birth and in the postpartum period with prenatal care, educational and therapeutic groups, employment enhancement services and substance abuse education. They stayed with their infants on-site until release into the community, which was facilitated by the programme through housing arrangements, coordination with social services and day care on release. This programme was not strictly in prison but perinatal outcomes in the WIAR group were compared to outcomes of women in prison before the programme existed, and to women in prison who were eligible for, but did not participate in WIAR. Therefore these studies were included because the comparison groups of pregnant women were in prison. When describing outcomes in prison under different models of care, we used only outcomes from women in the WIAR studies who were actually in prison (hence in the *PRISON* group). In the analysis comparing interventions to a comparison group, the intervention groups are the women in the WIAR programme (*PRISON++* intervention) and the comparison groups are the women in the standard prison (*PRISON)*.

## Describing outcomes in prison

There was little consistency in the reporting of outcomes: the largest number of studies reporting any one outcome was six. Many outcomes were reported in only one study, and these are not all reported here. Fourteen outcomes were reported in more than one study, enabling us to describe outcomes across studies, and where possible, compare outcomes in *PRISON+* to *PRISON* (Table 5). Sample sizes were often small, limiting our ability to detect statistical differences between groups, particularly for rare outcomes such as stillbirth, neonatal death, small for gestational age and APGAR score.

**Table 5:** Outcomes in *PRISON* and *PRISON+ groups*

Outcome	Studies in *PRISON* category			Studies in *PRISON+* category			All studies	
	Number of studies measuring outcome	Outcome (n/N)	Summary percentage (95 % CI)^a^	Number of studies measuring outcome	Outcome (n/N)	Summary percentage (95 % CI)^a^	Total number of studies measuring outcome	Overall summary percentage (95 % CI)^a^
Low birthweight	5	36/333	9 · 3 (6 · 2–12 · 4)	0	N/A	N/A	5	9 · 3 (6 · 2–12 · 4)
Caesarean^b^	3	37/137	26 · 5 (12 · 7–40 · 3)^b^	3	56/396	12 · 9 (8 · 3–17 · 6)^b^	6	N/A
Stillbirth	3	6/313	1 · 8 (0 · 3–3 · 3)	1	1/237	0 · 4 (0 · 0–1 · 2)	4	0 · 8 (0 · 0–1 · 5)
Neonatal unit admission	3	34/263	12 · 7 (8 · 7–16 · 8)	1	24/236	10 · 2 (6 · 3–14 · 0)	4	11 · 4 (8 · 6–14 · 2)
Inadequate prenatal care^b^	2	166/540	30 · 7 (26 · 8–34 · 6)^b^	1	34/221	15 · 4 (10 · 6–20 · 1)^b^	3	N/A
Preterm delivery^b^	2	24/119	19 · 0 (9 · 5–28 · 6)^b^	1	15/236	6 · 4 (3 · 2–9 · 5)^b^	3	N/A
Neonatal death	2	5/196	2 · 2 (0 · 1–4 · 2)	0	N/A	N/A	2	2 · 2 (0 · 1–4 · 2)
Small for gestational age	2	9/43, 0/34	N/A^c^	1	28/236	11 · 9 (7 · 7–16 · 0)	3	N/A
APGAR < 7 at 5 mins	2	5/76, 0/34	N/A^c^	1	0/236	0	3	N/A
Breastfeeding	2	1/34, 21/43	N/A^d^	1	3/5	60 · 0 (17 · 1–100)	3	N/A

^a^Summary measures created by taking results from individual studies, weighting by sample size and calculating summary statistic. Only presented if there is no significant heterogeneity between groups

^b^There is evidence of significant heterogeneity between *PRISON and PRISON+* groups (*p* = 0 · 005 for caesarean, *p* < 0 · 001 for inadequate prenatal care, *p* = 0 · 001 for preterm delivery), therefore a random effects model has been used

^c^No summary measure possible as only one non-zero result

^d^No summary measure possible as significant heterogeneity in results within *PRISON* category

Five studies, all in the *PRISON* category, reported low birth weight, with rates ranging from 6 to 17 % [16, 24, 26, 31, 32]. Mean birth weight was reported in six studies, three in the *PRISON* group with values of 3100 g, 3165 g and 3299 g [16, 24, 25], and three in the *PRISON+* group with values of 2495 g, 3153 g and 3299 g [21, 28, 29]. Only one study reported the associated standard deviation, making it impossible to summarise these figures [24]. Mean gestational age was reported in two studies: 38 · 8 weeks (standard deviation 2.2) in a *PRISON* study [16] and 39 weeks (no standard deviation reported) in a *PRISON+* study [29].

**Fig. 2:**
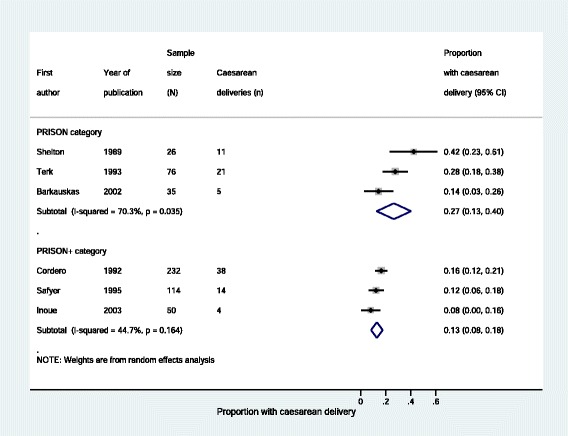
Caesarean deliveries to imprisoned women, stratified by level of care

**Fig. 3:**
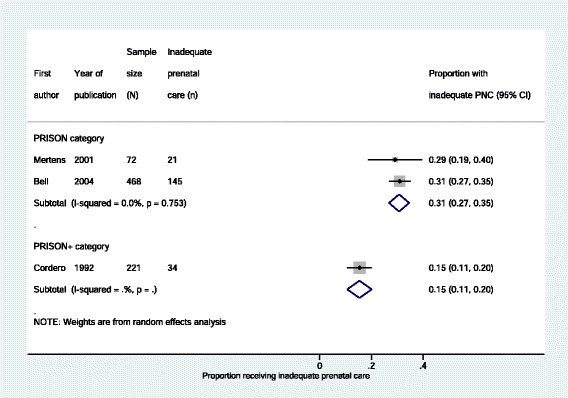
Imprisoned women receiving inadequate prenatal care, stratified by level of care

**Fig. 4:**
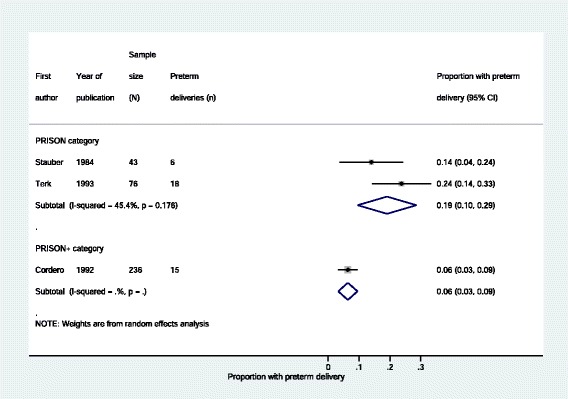
Preterm deliveries to imprisoned women, stratified by level of care

There was some evidence that rates of caesarean delivery (Fig. 2 and Table 5), inadequate prenatal care (Fig. 3 and Table 5) and preterm delivery (Fig. 4 and Table 5) were lower in women in prisons receiving enhanced prison care (*PRISON+)* compared to women in prisons receiving usual care (*PRISON).* For all these outcomes, the p-value for heterogeneity – using the I^2^ test – when comparing *PRISON* and *PRISON+* groups was less than 0 · 05.

“Inadequate prenatal care” was defined slightly differently in each of the three studies. Cordero defines it as women receiving less than six prenatal visits [21], Bell defines it using an “Adequacy of Prenatal Care Utilisation Index” which takes multiple factors into account [17], and Mertens calls it “Low antepartum care” without defining it more clearly [26].

**Fig. 5:**
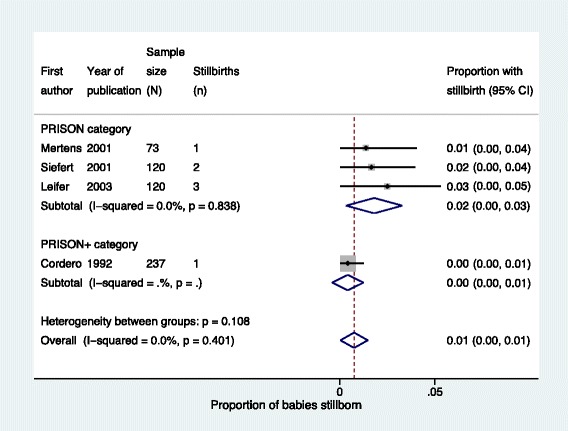
Stillbirths of babies born to imprisoned women, stratified by level of care

**Fig. 6:**
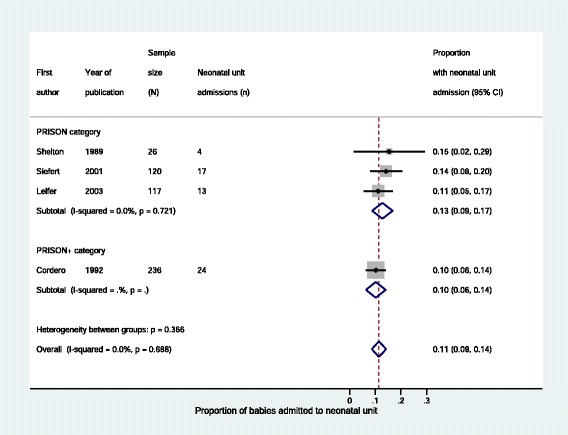
Neonatal unit admissions of babies born to imprisoned women, stratified by level of care

There was no significant difference in rates of stillbirth (Fig. 5 and Table 5) or neonatal unit admission (Fig. 6 and Table 5) between *PRISON* and *PRISON+* groups. For low APGAR score and small for gestational age it was not possible to compare outcomes between *PRISON* and *PRISON+* groups as there were not enough non-zero outcomes (Table 5). For breastfeeding rate, there was evidence of heterogeneity between the two studies in the *PRISON* group so we did not pool the data and could not compare outcomes in the *PRISON* and *PRISON+* groups (Table 5).

Women’s satisfaction with a particular prison intervention was measured in three studies. 5/5 women in one *PRISON+* programme felt that “contact with the programme was extremely positive” [27], and 14/14 women in another were “very satisfied” [29]. In Carlson’s *PRISON++ study,* 35/37 women said they would “go through the programme again” [18].

### Outcomes in intervention groups compared to comparison groups

Of the nine studies with useable comparison groups, five were in the *PRISON* category [17, 24, 26, 32, 33] and four were in the *PRISON++* category [16, 19, 23, 31]. We present the analysis separately for the *PRISON++* and *PRISON* studies as we did not deem the intervention groups to be similar enough to pool the results across those studies.

Of the five *PRISON* studies with comparison groups, three had population comparison groups [26, 32, 33], one had a disadvantaged comparison group [17], and one included both population and disadvantaged comparison groups [24]. Table 6 shows the outcomes of interest which were only reported in one study each, all of which had population comparison groups. There was no significant difference found between intervention and comparison groups for caesarean delivery, neonatal death, stillbirth, low APGAR score and small for gestational age. Women in the *PRISON* group were significantly less likely to breastfeed than population controls (OR 0 · 28, 95 % CI 0 · 14–0 · 56).

**Table 6:** Outcomes in *PRISON group* compared to population controls, for outcomes only reported in one study

Outcome	Intervention (n/N)	Comparison (n/N)	Odds ratio (95 % CI)
Caesarean section	21/76	26/117	1 · 34 (0 · 69–2 · 60)
Neonatal death	3/76	1/117	4 · 77 (0 · 49–46 · 7)
Stillbirth	1/73	189/15478	1 · 12 (0 · 16–8 · 13)
APGAR score < 7 at 5 minutes	5/76	0/117	18 · 1 (0 · 98–332)
Small for gestational age	9/43	28/172	1 · 36 (0 · 59–3 · 15)
Breastfeeding	21/43	133/172	0 · 28 (0 · 14–0 · 56)

**Fig. 7:**
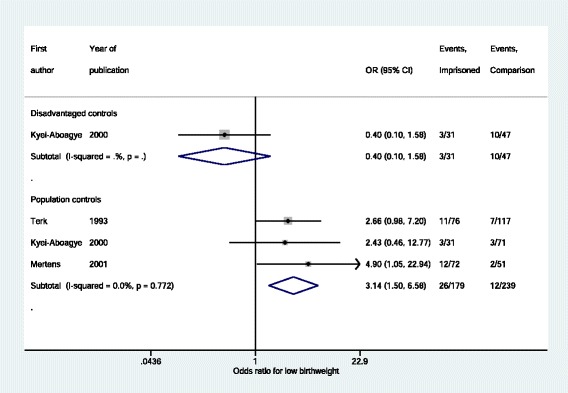
Low birthweight of babies born to women in *PRISON* intervention groups compared to controls, stratified by type of comparison group

Mean birth weight was reported in one study with both population and disadvantaged comparison groups [24]. The mean birth weight of babies born to the women in prison was not significantly different to those born to women in the population control group (standardised mean difference −0 · 19, 95 % CI −0 · 61 to 0 · 23) or the disadvantaged control group (standardised mean difference 0 · 39, 95 % CI −0 · 07 to 0 · 85). Low birth weight was found to be significantly more common in imprisoned women compared to population controls (OR 3 · 14, 95 % CI 1 · 50–6 · 58) but no difference was found comparing imprisoned women to disadvantaged controls (OR 0 · 40, 95 % CI 0 · 10–1 · 58) (Fig. 7). Inadequate prenatal care was reported in two *PRISON* studies [17, 26] and was significantly more likely in intervention groups (summary OR 1 · 87, 95 % CI 1 · 25–2 · 81) than in comparison groups, which were one group each of disadvantaged and population controls. Preterm delivery rates were significantly higher among imprisoned women in two *PRISON* studies compared to population controls (summary OR 2 · 06, 95 % CI 1 · 12–3 · 79) [32, 33].

The four *PRISON++* papers examining two residential interventions compared outcomes in the intervention groups to disadvantaged controls who were in fact themselves in prison [16, 19, 23, 31]. The following outcomes were only reported in one paper each: caesarean delivery, neonatal death, stillbirth, low APGAR scores, NICU admission, small for gestational age, breastfeeding and mean gestation. There was no significant difference in these outcomes comparing intervention women to comparison women (Table 7).

**Table 7:** Outcomes in *PRISON++ group* compared to disadvantaged controls, for outcomes only reported in one study

Outcome	Intervention (n/N)	Comparison (n/N)	Odds Ratio (95 % CI)
Caesarean section	4/37	5/35	0 · 73 (0 · 18–2 · 96)
Neonatal death	0/45	2/120	0 · 52 (0 · 02–11 · 1)
Stillbirth	0/45	2/120	0 · 52 (0 · 02–11 · 1)
APGAR score < 7 at 5 minutes	1/37	0/34	2 · 84 (0 · 11–72 · 0)
Neonatal unit admission	1/45	17/120	0 · 14 (0 · 02–1 · 07)
Small for gestational age	1/32	0/34	3 · 29 (0 · 13–83 · 6)
Breastfeeding	7/36	1/34	7 · 45 (0 · 87–64 · 1)
Mean gestation^a^	Mean 38 · 9, SD 1 · 7	Mean 38 · 8, SD 2 · 2	0 · 05 (−0 · 41 to 0 · 52)

^a^standardised mean difference instead of odds ratio presented

**Fig. 8:**
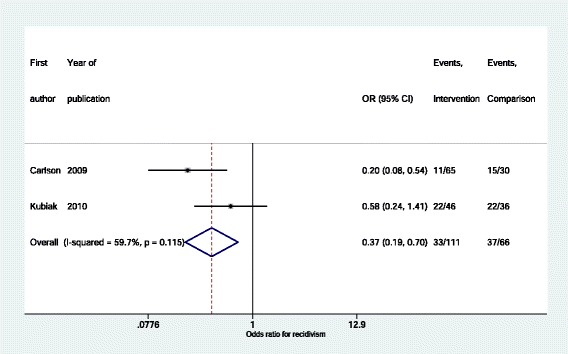
Recidivism of women in *PRISON++* intervention groups compared to disadvantaged controls

Low birth weight was reported in two *PRISON++* studies [16, 31] and was not significantly different between intervention and comparison groups (summary OR 1 · 22, 95 % CI 0 · 42–3 · 55). Recidivism rates were reported in two studies [19, 23] – each one examining a different intervention – and there is evidence that the *PRISON++* interventions reduced recidivism compared to women in prison who did not receive the intervention (summary OR 0 · 37, 95 % CI 0 · 19–0 · 70) (Fig. 8). Recidivism was defined in one study as “confined for any offence post-birth” and was measured using a large administrative database up to the year 2008 which was between eight and ten years after the birth of the women’s babies [23]. In the other it was defined as “returned to the facility for violating parole or committing a new crime”, measured from the facility records, and was sought up to the year 2007 which was between three and 13 years after the birth of the babies [19]. We considered these two measures of recidivism similar enough to pool the data across the two studies.

## Discussion

This is the first systematic review to examine perinatal health care services for pregnant women. We reviewed comprehensively the research literature and examined the evidence for the effectiveness of models of care. We categorised the perinatal health care provided in prison into three distinct groups we termed *PRISON*, *PRISON+* and *PRISON++* depending on the level of care provided to the imprisoned women. There was some evidence that women in prisons with increased perinatal care provision had improved maternal and perinatal outcomes; women in prisons receiving enhanced prison care, *PRISON+,* appeared to be less likely to have inadequate prenatal care, a preterm delivery or a caesarean delivery when compared to women in prisons receiving usual prison care. The two *PRISON++* interventions, that is, interventions which included not only enhanced care in prisons and co-residence with children after birth, but also coordination of community care on release, demonstrated reductions in recidivism rates over the 10 years following release when compared to women in the same prisons who did not take part in the intervention. This finding suggests that a long-term outcome can be improved when interventions are designed in a way that supports women beyond just their time in prison. There is evidence that children of incarcerated parents are more likely to experience a range of negative outcomes than children of similar socioeconomic backgrounds who do not have an incarcerated parent [87]; thus reduced recidivism in the years following birth could impact positively on the lives of both mother and child.

One of the most striking findings of this review was the lack of data and in particular, a lack of high quality studies. Thirty five studies were excluded because although examining specific interventions, they did not quantify any outcomes. This suggests that that there is a paucity of meaningful data evaluating the quality and impact of programmes, even where the programmes exist. Of those studies that were included, the quality was variable (Table 4). Risk of selection bias was assessed as generally high among the prison populations. In nine studies it was unclear how prisoners were selected [17, 20–22, 25, 27, 30–32], and in eight there was a high risk of selection bias [16, 18, 19, 23, 24, 26, 28, 31], largely because of strict eligibility criteria for entering the intervention programmes which excluded a high proportion of imprisoned pregnant women. If or when critical justice reform happens in the US it will be vital that women with violent charges are not excluded from enhanced programmes, as otherwise very few women will be eligible to participate. Assessing performance bias, that is, whether intervention or comparison groups were exposed to care other than that described, was almost impossible as authors did not comment on this. There was also a high risk of selective outcome reporting, with eight studies reporting outcomes that were not described in their methods or omitting to report outcomes included in the aims [18, 19, 22, 25, 30–32]. The overall risk of outcome measurement bias was low in nine studies [17, 20–22, 24, 25, 28, 29, 32], high in six studies [16, 18, 19, 23, 27, 30], unclear in two [31, 33] and mixed in one [26]. Risk of measurement bias was high for both measures of recidivism. There were no other outcomes that had consistently high risk of bias. In all studies with comparison groups except one [23]; it was felt that there were comparable methods measuring outcomes in intervention and comparison groups. All the studies were observational and thus there remains possibility of uncontrolled confounding accounting for some of the observed differences.

We recognise the difficulties of conducting research in prisons. These are challenging environments where, for example, prison regimes make it difficult to access women, there are unique ethical considerations and research funding opportunities are limited. However, with careful planning and engagement of all stakeholders, it is possible for large scale and high quality research to be conducted.

Length of stay in prison has been demonstrated to have an effect on perinatal outcomes [88], and it is a limitation of our study that we were not able to adjust outcomes based on timing of entry into prison and length of imprisonment of pregnant women. This information was only available in one study [32], reflecting again the lack of high quality studies. Related to this is the difference between prisons (longer term inmates) and jails (shorter term inmates) in the US. The *PRISON* and *PRISON+* studies from the US were based in a mixture of prisons and jails. All *PRISON++* studies and their comparison groups were in US prisons.

Previous systematic reviews have shown that imprisoned women are a high risk obstetric group [10] and that imprisoned women may have improved pregnancy outcomes compared to similarly disadvantaged women outside prison [12]. One possible explanation for these improved outcomes - is that prison provides protection from the disarray of women’s lives outside prison, which includes enabling them to access antenatal care. The findings of this latest review, although limited by the quality of the included studies, suggest that greater health and social care input leads to improved outcomes relating to adequate prenatal care, preterm delivery and caesarean delivery; and that programmes providing longer term support can reduce recidivism. These results do not endorse the imprisonment of pregnant women. They focus on a limited set of outcomes and do not examine the wider psychosocial or ethical aspects of imprisoning pregnant women. If women need to be incarcerated, they should be provided with excellent care in a correctional facility. If they do not need to be incarcerated they should be supported in the community. There are no clinical trials which compare imprisonment to enhanced community support programmes like one of the *PRISON++* programmes in this review [16, 23, 31], and it is possible that there would be better perinatal and long-term outcomes for women and children who are supervised in the community. There is some limited evidence that antenatal care programmes targeting specific vulnerable groups are effective [89]. Specific models of care appeared to confer benefits on particular vulnerable groups such as drug users, socioeconomically deprived and teenage pregnant women [90–96]. Again however, most data related to short-term outcomes, and it is also important to investigate long-term outcomes given the potential benefits not just to the mother and child but also wider society.

We used an extensive search strategy and were able to locate relevant studies that had not been published in scientific journals. However, most studies were located in the US, limiting generalizability, and we particularly note the absence of any information from prisons in low and middle income countries, and the absence of studies in juvenile facilities. This study was also limited by the poor quality of the component papers. There was little consistency in the reporting of outcomes and very few measured beyond the postnatal period. Outcomes were not always defined consistently and another limitation is that we have pooled some data comparing outcomes across studies where the outcomes have been defined slightly differently, for example for recidivism and inadequate prenatal care. The largest number of studies reporting any one outcome was only six and many outcomes were reported in only one study. This systematic review provides evidence of the need for a minimum set of outcomes to be reported in future studies looking at the perinatal health care of imprisoned pregnant women.

For many outcomes there were small sample sizes and only a few cases in each group, which limits our power to detect significant differences between groups. Studies with low power have a reduced chance of detecting a true effect but also a reduced likelihood that a statistically significant result reflects a true effect. Future studies should use adequate sample sizes to detect significant differences between groups. For example, to differentiate between stillbirth rates of 0.5 % in one group and 1 % in another, 4,600 in the cohort and 4,600 in the comparison group would be needed. To detect a reduction in recidivism rate from 50 to 30 %, 100 in the cohort and 100 in the comparison group are needed.

There was some heterogeneity regarding the intervention groups, particularly in the *PRISON++* group. One *PRISON++* intervention was a nursery programme within the prison, and the other was a secure community-based residential facility for women to reside during pregnancy and after birth with their child. However, they both provided alternative accommodation to the usual prison accommodation, enabled mother and child to reside together, linked to community resources on release, and were compared to women in prison who were given no specific support during their pregnancies. We therefore decided to pool the data across the two studies measuring recidivism.

Perinatal care in prison is an important opportunity for health professionals to engage this vulnerable yet accessible population with potentially significant impacts on the long-term health of both mother and baby. Of the main modifiable risk factors during pregnancy for future child health (tobacco, alcohol, obesity, diet, illicit drug use, mental illness, low socio-economic status and psychosocial stress) [97], most could be targeted through a comprehensive perinatal care programme for pregnant prisoners. There are some interventions designed specifically for pregnant prisoners but very few of these support women beyond the immediate postnatal period, and they are not being adequately evaluated. Despite WHO’s 2009 declaration that current arrangements for dealing with the health of women in prison fall far short of what is required by human rights [2], we are currently missing the opportunity to improve both the short and longer-term health of these women and their children.

Properly designed programmes with rigorous evaluation are needed as a matter of urgency so that those commissioning and providing services to these high risk women can ensure the delivery of evidence-based comprehensive perinatal care for imprisoned pregnant women. The design of such studies should be informed by the UK’s Medical Research Council guidelines on complex interventions [98], should not be confined to the US, the UK and Germany, and should include long-term follow up with outcomes agreed by an expert panel and supported by the literature, as outlined by the COMET initiative to develop Core Outcome Sets [99]. Action is also needed to highlight and encourage political action on this important public health issue.

## Conclusion

This is the first systematic review examining perinatal health care services for imprisoned pregnant women. Our findings suggest that increased perinatal care services for these women can improve both short and long-term outcomes. However, there is a paucity of data on the perinatal outcomes of imprisoned women and models of care, where they exist, are not being evaluated. Properly designed programmes with rigorous evaluation are needed so that we can better address appropriately the health needs of this vulnerable population. The costs to mothers, children and to society of failing to address these important public health issues are likely to be substantial.
